# Thoracic endovascular aortic repair for avulsion of aortic branches in a trauma patient requiring resuscitative thoracotomy: a case report

**DOI:** 10.1186/s40792-022-01427-4

**Published:** 2022-04-19

**Authors:** Atsushi Tanikawa, Takeaki Sato, Motoo Fujita, Chieri Tsuchiya, Ken Katsuta, Yusuke Suzuki, Kiichiro Kumagai, Yoshikatsu Saiki, Shigeki Kushimoto

**Affiliations:** 1 Department of Emergency and Critical Care Medicine, Tohoku University Hospital, 1-1 Seiryo-machi, Aoba-ku, Sendai, 980-8574 Japan; 2 Division of Cardiovascular Surgery, Tohoku University Graduate School of Medicine, 1-1 Seiryo-machi, Aoba-ku, Sendai, 980-8574 Japan; 3 Division of Emergency and Critical Care Medicine, Tohoku University Graduate School of Medicine, 1-1 Seiryo-machi, Aoba-ku, Sendai, 980-8574 Japan

**Keywords:** Trauma, Resuscitation, Thoracotomy, Aorta, Vascular injury, Endovascular procedures

## Abstract

**Background:**

Resuscitative thoracotomy is a lifesaving procedure for trauma patients that are hemodynamically unstable. Cross-clamping of the descending thoracic aorta is an essential procedure performed during resuscitative thoracotomy in patients with impending cardiac arrest. Although complications related to resuscitative thoracotomy have been reported, there is no report on avulsion of aortic branches related to cross-clamping of the descending aorta and its appropriate management.

**Case presentation:**

We present the case of a 42-year-old woman who sustained blunt trauma due to an accidental fall. The patient was hemodynamically unstable and required resuscitative thoracotomy with cross-clamping of the thoracic aorta. However, hemorrhage from avulsion of aortic branches related to aortic cross-clamping was identified. Initially, transcatheter arterial embolization was attempted to achieve hemostasis; however, when that proved ineffective, thoracic endovascular aortic repair was performed, which resulted in successful hemorrhage control without any sequelae.

**Conclusions:**

Thoracic endovascular aortic repair may be a management option for aortic branch avulsion due to cross-clamping of the descending aorta during resuscitative thoracotomy.

## Background

Resuscitative thoracotomy (RT) is a lifesaving procedure for trauma patients that are hemodynamically unstable [1–4]. Cross-clamping of the descending thoracic aorta is an essential procedure performed during RT in patients with impending cardiac arrest to maintain cerebral and cardiopulmonary circulation. Although complications related to RT, including lacerations of the heart, aorta, and lung, have been reported [5, 6], their incidence, appropriate management, and impact on outcomes have not been clarified. In addition, avulsion of aortic branches related to cross-clamping of the descending aorta and its management have not been reported. Therefore, this report aimed to present a case of successful management of avulsion of aortic branches related to cross-clamping of the descending aorta during RT using thoracic endovascular aortic repair (TEVAR).

## Case presentation

A 42-year-old woman fell from a height of 15 m. During pre-hospital evaluation, her Glasgow Coma Scale (GCS) score was 14, the right lower extremity was deformed, and the radial artery pulse was weakly palpable. On arrival at our institution, her GCS score was 13, blood pressure was 60/30 mmHg, heart rate was 140 bpm, and respiratory rate was 50 breaths per minute. As part of initial resuscitation, resuscitative endovascular balloon occlusion of the aorta (REBOA) was performed to prevent further hemodynamic deterioration due to lower extremity and pelvic traumas. During catheterization, however, her systolic blood pressure decreased to 30 mmHg, and she deteriorated to near imminent cardiac arrest.

To prevent the aggravation of hypotension and risk of cardiac arrest, we performed RT and cross-clamping of the descending aorta through the left 4th intercostal space in the supine position. At the time of RT, a small hemopneumothorax and multiple rib fractures were observed. Hemopericardium or mediastinal hematoma was not identified. Her systolic blood pressure increased to approximately 90 mmHg after cross-clamping of the aorta, and cardiac arrest was averted; therefore, REBOA was completed, and direct aortic clamping was converted to balloon occlusion of the aorta to perform contrast-enhanced computed tomography (CT) under controlled balloon occlusion of the aorta.

**Fig. 1:**
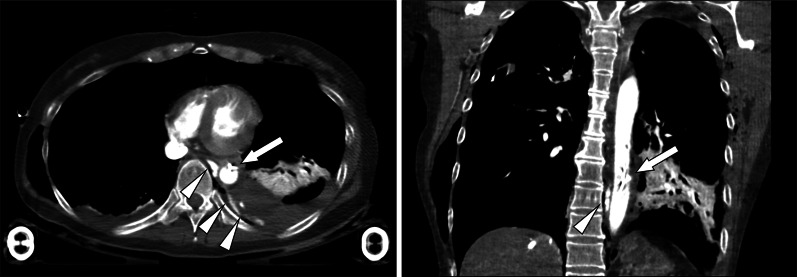
Computed tomography findings after aortic cross-clamping. Contrast-enhanced computed tomography showing an endovascular balloon in the thoracic aorta (arrow) and extravasation of contrast medium adjacent to the descending aorta (arrowhead)

**Fig. 2:**
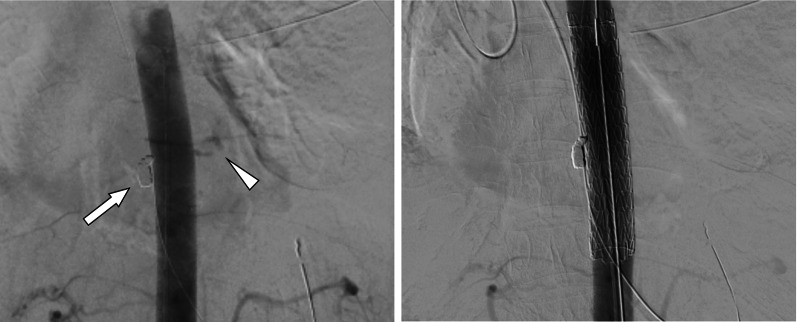
Computed tomography findings after transcatheter embolization and thoracic endovascular aortic repair. Digital subtraction angiography showing the coil placed during transcatheter arterial embolization (arrow) and extravasation of contrast medium adjacent to the descending aorta (arrowhead) (right). After deploying the thoracic aortic stent-graft, the extravasation disappeared (left)

Contrast-enhanced CT showed bilateral pneumothorax, pulmonary contusion, bilateral multiple rib fractures, pelvic fracture, and right femoral diaphyseal fracture. Extravasation of contrast media from the descending aorta was also observed (Fig. 1). Based on the initial radiological findings of the left intrapleural cavity and mediastinum, avulsion of one or more aortic branches due to cross-clamping of the aorta during RT was suspected. Although her hemodynamic status improved after transcatheter arterial embolization of the aortic branches with coils, she developed hypovolemic shock after 6 h despite continuous blood transfusion. Repeat CT showed extravasation adjacent to the descending aorta. Therefore, we decided to perform TEVAR for controlling the hemorrhage occurring at the site of avulsion of the aortic branches. The stent-graft (Conformable GORE TAG 21 × 100 mm, Gore Medical, Newark, DE, USA) was deployed to adequately cover the avulsion of the aortic branches, and extravasation from the aorta eventually disappeared (Fig. 2). Complications of TEVAR, including paraplegia and other thrombo-ischemic events, did not occur. She was discharged on the 59th hospital day.

## Discussion

This case highlights the efficacy of TEVAR in controlling hemorrhage from the avulsion of aortic branches related to cross-clamping of the descending aorta performed during RT.

RT is considered a last resort in the management of patients with major trauma. It is performed in patients with penetrating or blunt thoracic trauma or exsanguinating abdominal injury, if appropriate resources are available, for definitive injury management. Indications for RT include direct cardiac compression, cardiac tamponade, treatment of cardiac or thoracic injury, prevention of air embolism, and cross-clamping of the aorta [1]. An aortic clamp may be lifesaving when performing acute resuscitation for severe hemorrhagic shock. Occlusion of the thoracic aorta decreases blood loss and results in increased blood pressure, which improves perfusion to the heart, lungs, and brain without sacrificing blood flow to the abdomen, pelvis, and lower extremities. Aortic cross-clamping is an essential procedure for critical trauma patients with unstable hemodynamics and risk of imminent cardiac arrest.

RT is a favorable method for patients facing imminent cardiac arrest due to cardiac tamponade and tension pneumothorax that can lead to chest trauma. For patients with hemorrhagic shock in non-thoracic trauma without imminent cardiac arrest, on the other hand, REBOA is more favorable option and less invasive procedure than RT. In our case, we first performed REBOA, since the patient had non-thoracic trauma without imminent cardiac arrest. However, the procedure of REBOA resulted in further progression of shock; therefore, REBOA was switched to aortic cross-clamping with RT to prevent cardiac arrest. It is true that cross-clamping of the aorta with RT is a more invasive procedure than balloon occlusion of the aorta in REBOA. To prevent cardiac arrest, however, it is more important to make decisions, such as switching from REBOA to RT or initiating with RT in patients who are deteriorating rapidly.

It is important to recognize and comprehend the complications associated with RT. Some complications of RT have been reported in previous studies, such as lacerations of the heart, aorta, and avulsion of the aortic branches supplying blood to the mediastinum [5–7]. However, to our knowledge, there are no studies on the pathophysiology, incidence, and appropriate management of these complications and the percentage of these complications and related survival outcomes are unclear.

The complication reported in this case could have been prevented had the aortic clamp been applied with periaortic dissection under direct visualization of the aorta. However, it may be difficult to perform these procedures under emergent conditions. In this case, hemorrhage from the avulsion of aortic branches related to cross-clamping of the aorta was successfully controlled with TEVAR. To the best of our knowledge, this is the first report on TEVAR as a therapeutic modality for achieving hemostasis at the site of avulsion of aortic branches.

## Conclusions

TEVAR may be a suitable management option for avulsion of aortic branches related to cross-clamping of the descending aorta in patients requiring RT.

## Data Availability

Not applicable.

## Abbreviations

RT: Resuscitative thoracotomy
TEVAR: Thoracic endovascular aortic repair
GCS: Glasgow coma scale
REBOA: Resuscitative endovascular balloon occlusion of the aorta
CT: Computed tomography

## References

[1] Cothren CC, Moore EE (2006). Emergency department thoracotomy for the critically injured patient: objectives, indications, and outcomes. World J Emerg Surg.

[2] Boddaert G, Hornez E, De Lesquen H, Avramenko A, Grand B, MacBride T (2017). Resuscitation thoracotomy. J Visc Surg.

[3] Narvestad JK, Meskinfamfard M, Søreide K (2016). Emergency resuscitative thoracotomy performed in European civilian trauma patients with blunt or penetrating injuries: a systematic review. Eur J Trauma Emerg Surg.

[4] Working Group, Ad Hoc Subcommittee on Outcomes, American College of Surgeons. Committee on Trauma. Practice management guidelines for emergency department thoracotomy. Working Group, Ad Hoc Subcommittee on Outcomes, American College of Surgeons-Committee on Trauma. J Am Coll Surg. 2001;193:303–9. 10.1016/s1072-7515(01)00999-1 11548801

[5] Burlew CC, Moore EE, Feliciano DV, Mattox KL, Moore EE, Ball CG, Kozar R, Alam HB (2020). Resuscitative thoracotomy. Trauma.

[6] Suliburk JW (2012). Complications of emergency center thoracotomy. Tex Heart Inst J.

[7] Aseni P, Rizzetto F, Grande AM, Bini R, Sammartano F, Vezzulli F (2021). Emergency department resuscitative thoracotomy: indications, surgical procedure and outcome. A narrative review. Am J Surg.

